# Selected Health Status Measures of Children from US Immigrant Families

**DOI:** 10.1155/2013/164757

**Published:** 2013-07-08

**Authors:** Stella M. Yu, Sue C. Lin, Terry Adirim

**Affiliations:** ^1^Maternal and Child Health Bureau, Health Resources and Services Administration, Department of Health and Human Services, Room 18A-55, 5600 Fishers Lane, Rockville, MD 20857, USA; ^2^Office of Quality & Data, Bureau of Primary Health Care, Health Resources & Services Administration, Rockville, MD 20857, USA; ^3^Office of Special Health Affairs, Health Resources & Services Administration, Rockville, MD 20857, USA

## Abstract

Using the 2007 National Survey of Children's Health (*N* = 91,532), we studied the relationship between the joint effects of immigrant family type (foreign-born children, US-born children/one foreign-born parent, US-born children/both foreign-born parents, and US-born children/US-born parents) and race/ethnicity on various health measures (parent-reported physical and dental health, obesity/overweight, breast-feeding, school absence, injury, and chronic condition). We used weighted logistic regression to examine the independent effects of the 12-level joint variable on various health status measures while controlling for confounding factors. Overall, nearly one-third of families with both foreign-born parents were poor, and one-quarter of the parents in these households did not complete high school. Compared with non-Hispanic White US-born children, multivariable analyses indicate that all Hispanic children have higher odds of obesity, poor physical and dental health, with Hispanic foreign-born children 7 times as likely to report poor/fair physical health. Most children of immigrant parents were more likely to have been breast-fed and less likely to miss school more than 11 days. Child age and household poverty status were independently associated with most of the health status measures. Combined race/ethnicity and immigrant family type categories have heterogeneous associations with each health outcome measure examined. Culturally competent interventions and policies should be developed to serve these expanding communities.

## 1. Introduction

Children from US immigrant families, who are defined as individuals under the age of 18 in families with at least one foreign-born parent, comprised nearly one-quarter of all US children in 2010 [1]. In USA, immigrant families have been differentiated by the following types: those with foreign-born children, US-born children with both foreign-born parents, and US-born children with one foreign-born parent. Many of these children live in households with low incomes, have parents with low education levels and limited English proficiency, interact less often with their parents, and use less health care benefits than children of natives [2]. Immigrant families are driving rapid population increases and growing racial and ethnic diversity in local communities and school districts across the country [3]. This significant demographic shift presents a unique set of social and economic challenges for access to health care, oral health, and health promotion outcomes. 

Differential health care access and use have been found in USA and Canada for specific immigrant family types [4–6]. From 2003 to 2006, an estimated 30.6% of direct medical care expenditures for African American, Asian American, and Hispanics was excess costs caused by health inequalities [7]. Immigrant parents with limited English proficiency are at high risk of alienation from health care systems and support services that are available to low-income and other vulnerable populations in the United States [8, 9]. For the nearly 14% of all US children living in non-English-primary-language households, multiple factors including the race and ethnicity of their families, cultural beliefs about health, trust of the US health care system, and the family resources brought to the US are all related to barriers to health service access [8, 10, 11]. Despite heterogeneity in health care access and utilization, some children from immigrant families have demonstrated better than expected health status [12, 13]. Both the American Academy of Pediatrics' Committee on Community Health Services and Vision of Pediatrics 2020 Task Force recognized the unique and complex medical and psychosocial risks faced by immigrant children [14, 15]. Most studies to date on immigrant children have focused on health care access issues, while less is known about their health status.

Previous studies have reported on immigrant children's health measures that were often dependent on provider diagnosis, thus excluding children not engaged in the health care system. A number of other studies have reported on risk and protective factors on limited measures of children's health in smaller immigrant populations. Breast-feeding was found to be less prevalent in disadvantaged populations [16]. Acculturation was associated with lower breastfeeding rates among both Hispanic and non-Hispanic women [17–19]. Immigrant patterns in childhood obesity and overweight vary substantially by ethnicity and generational status with increased risk from longer duration of residence [20–22]. Immigrant children generally had lower physical inactivity, lower sports participation levels, and lower obesity and overweight prevalence and body mass index than native children [23, 24]. Significant disparities were also reported in oral health status, in the number receiving the recommended number of dental visits based on American Academy of Pediatric Dentistry (AAPD) Bright Futures recommendations, as well as referrals for dental conditions and parental oral health knowledge [25–27]. For school attendance, Asian American children were less likely to miss school because of illness or injury or have learning disabilities compared with non-Hispanic whites [10].

To further investigate the heterogeneity in the health status of immigrant children in one single national probability sample, this study examines the relationship between the joint effects of immigrant family type and race/ethnicity and selected objective health measures using data from the 2007 National Survey of Children's Health (NSCH). Our study seeks to advance the understanding of the complex relationships and independent factors contributing to the attainment of optimal growth, development, and well-being of children from US immigrant families. 

## 2. Methods

The 2007 NSCH was conducted by the Centers for Disease Control and Prevention's National Center for Health Statistics (NCHS), with funding and direction from the Health Resources and Services Administration's Maternal and Child Health Bureau [28–30]. The purpose of the survey was to provide national and state-specific prevalence estimates for a variety of children's health and well-being indicators [28, 29]. The NSCH included information on children's physical and mental health, health care, and social well-being [28]. The survey also included an extensive array of questions about the family, including parental health, stress and coping behaviors, family activities, and parental concerns about their children, as well as questions about neighborhoods [28, 29].

The 2007 NSCH was a telephone survey conducted between April 2007 and July 2008, with a total sample size of 91,642 children from birth through 17 years of age, including a sample of about 1800 children per state [28, 29]. A random-digit-dial sample of households with children <18 years of age was selected from each of the 50 states and DC. One child was selected from all children in each identified household to be the subject of the survey. Interviews were conducted in English, Spanish, and 4 Asian languages: Mandarin, Cantonese, Vietnamese, and Korean [28, 29]. The respondent was the parent or guardian who knew most about the child's health status and health care. The interview completion rate, measuring the percentage of completed interviews among known households with children, was 66.0%. The overall response rate at the national level was 46.7% [29]. Substantive and methodological details of the survey are described elsewhere [29]. The NCHS Research Ethics Review Board approved all data collection procedures.

The independent variable was a 12-level composite variable formed by combining the child's race/ethnicity and immigrant family type. The race/ethnicity categories included Hispanic, non-Hispanic White (NHW), non-Hispanic Black (NHB), and all other ethnic groups. The immigrant family type consisted of the following: foreign-born (FB) child with both immigrant parents, US-born (USB) child with both immigrant parents, USB child with one immigrant parent, and the USB child with both USB parents. NHW USB children were used as the comparison group as they comprised more than 60% of the sample. 

The health status measures were selected from the ones that did not require health care provider input, with the exception of chronic condition. Several of these measures were among the national indicators of the child as reported in the Health and Well-Being of Children: A Portrait of States and Nation 2007 [30]. The health measures for this study included parent-reported child health status, oral health status, obesity/overweight, breastfeeding, missed school days, injury, and prevalence of at least one chronic condition. 

Parent-reported and child health and oral health status was dichotomized into excellent/very good/good or fair/poor. The obese/overweight measure was determined by the body mass index (BMI) for age standards. BMI was calculated by parental report of weight in kilograms divided by the square of height in meters for children aged 10–17. Breastfeeding was ascertained by a question asking parents of children aged 0–5 years if the child was ever breast-fed or fed breast milk. For missed school days, the NSCH inquired parents of school-aged children (aged 6–17 years) with regard to how many days of school their children had missed due to illness or injury during the past year. Within the early childhood section, the injury measure was based on parent report of whether their child had been injured and required medical attention during the past month. For all children aged 0–17 years, parents were asked if their child had one or more of the 16 chronic conditions listed such as behavioral and developmental problems, ADHD, autism spectrum disorder, asthma, and diabetes.

We considered the following variables as covariates in our analyses: child's age (0–3, 4–7, 8–11, 12–14, and 15–17 years), gender, and household poverty status (FPL) measured as a ratio of family income to poverty threshold (<100%, 100%–199%, 200%–399%, and ≥400%).

### 2.1. Statistical Analysis

With a final analytic sample of 91,532 children, chi-square analyses were used to test for sociodemographic and health measures differences across immigrant family types. Separate logistic regression analyses were used to examine the joint effects of race/ethnicity and immigrant family type on health outcomes adjusting for children's age, gender, and FPL. Educational level was not included as a covariate due to its collinearity with income. Income has been shown to be a better predictor of SES for immigrants since education credentials from foreign countries often result in underemployment in USA [31]. Primary language was also collinear with the primary independent variable and was not included in the final models. Adjusted odds ratios (OR) and 95% confidence intervals (CI) were computed by using the beta coefficients and standard errors obtained from the multivariable logistic analyses. To account for the complex sample design involving stratification, clustering, and multistage sampling of the NSCH, SUDAAN version 10.0 was used to conduct the statistical analyses [32]. Taylor series linearization methods were applied for variance estimation as recommended.

## 3. Results

Table 1 shows the demographic and socioeconomic characteristics by immigrant family type. The sample included 2600 FB children, 4622 USB children with both FB parents, and 2543 USB children with one FB parent. The comparison group consisted of 81767 USB children with USB parents. Significant associations were found between immigrant status and each of the demographic characteristics with the exception of gender (*χ* ^2^, *P* value < 0.0001). Regarding children's race and ethnicity, the Hispanic group had the highest percentage (57%) of FB children and USB children with both FB parents. In addition, the percentage of “poor” children with both FB parents was substantially higher than USB children with one or both FB parents. Moreover, about one-third of the parents from both FB parent households had less than a high school education. Parents of USB children with one FB parent had the highest percentage of high school graduates. Over 89% of the USB children with one FB parent group spoke English as the primary language at home versus 28% for USB children with both FB parents and 36% for FB children. 

Table 2 shows the selected health measures of children by race/ethnicity and immigrant family type. Hispanic FB children had the highest percentage of reported fair/poor health (17%) and dental health (37%). Overall, Hispanic and non-Hispanic Black (NHB) children had higher rates of obese/overweight. Within “all other” ethnic groups, USB children with USB parents have the highest percentage of being obese/overweight. In general, children with one or both FB parents were more likely to be breast-fed than their peers with USB parents in the Hispanic, NHW, and NHB groups. Nearly 50% of NHB children with USB parents have never been breast-fed as compared to only 8.9% of children with one or both FB parents. Within each racial/ethnic group, a higher percentage of children with USB parents missed 11+ school days than their peers from other immigrant family types. The lowest percentage of injury was found in USB children with both FB parents from Hispanic and “all other” ethnic groups. Children of USB parents in each racial/ethnic group had the highest percentage of reporting at least one chronic condition. 

Table 3 shows results of the multivariable analysis on the association between race/ethnicity immigrant family type and selected health measures, after controlling for age, gender, and FPL. Compared with NHW children, Hispanic children from all family types were significantly more likely to be in fair/poor health. In particular, Hispanic FB children were almost seven times more likely to be in fair/poor health than NHW children (OR = 6.88, 95% CI 4.17, 11.37). Children from “all other” ethnic groups with one FB parent were least likely to be in fair/poor health (OR = 0.11, 95% CI 0.02, 0.50). When examining differences across age groups, older children were more likely to be in fair/poor health in age range of 6–8 years compared to children aged 0–3 years (4–7 years: OR = 2.36, 95% CI 1.69, 3.30; 8–11 years: OR = 2.71, 95% CI 1.99, 3.67; 12–14 years: OR = 3.57, 95% CI 2.57, 4.96; and 15–17 years: OR = 3.21, 95% CI 2.36, 4.36, resp.). With respect to FPL, poor children were five times more likely than the children at or above 400% to be in fair/poor health (OR = 5.09, 95% CI 3.50, 7.40). Similarly, near-poor children were close to three times as likely to be reported in fair/poor health (OR = 2.77, 95% CI 1.87, 4.10). 

Hispanic, NHB, and “all other” ethnic groups had higher odds to be in fair/poor dental health compared to NHWs. In particular, Hispanic FB children with both FB parents were over ten times as likely to be in fair/poor dental health (OR = 10.10, 95% CI 7.22, 14.12). Older children had higher odds of being in fair/poor dental health as compared to the age 3–5 group. Females were less likely to be in fair/poor dental health. In addition, poor children were more than seven times as likely to be in fair/poor dental health. Less affluent children were more likely to have fair/poor dental health than the highest income group. 

Both Hispanic and NHB children were more likely than NHW children from all family types to be obese/overweight. The children from “all other” ethnic groups with at least one FB parent (mostly Asians) were the least likely to be obese/overweight. Compared to children >400% of FPL, those from households with FPL <100%, 100–200%, and 200–400% all had higher odds of being obese/overweight (OR = 2.38, 95% CI 1.99, 2.85; OR = 1.93, 95% CI 1.65, 2.26; and OR = 1.47, 95% CI 1.29, 1.68, resp.). 

Almost all groups had lower odds of having never been breast-fed when compared to NHW children of USB Parents. The only exception was among NHB children with USB parents where they were twice as likely to have never been breast-fed. Lower household income does appear to be associated with higher odds of being never breast-fed. 

Compared with NHWs, most groups were less likely to have missed 11+ school days, with the exception of Hispanic USB children of USB parents having slightly higher odds of missing school 11+ days (OR = 1.22, 95% CI 0.88, 1.70). As children grew older, the odds of missing school were also higher. Children from households with FPL below 100%, 100–200%, and 200–400% had higher odds of missing 11+ school days compared to children from wealthier households (OR = 2.62, 95% CI 2.00, 3.43; OR = 1.92, 95% CI 1.49, 2.48; and OR = 1.34, 95% CI 1.03, 1.73, resp.). Hispanic and “all other” ethnic groups USB children with both FB parents were substantially less likely to report injury requiring medical care compared to NHW, USB children with USB parents (OR = 0.25, 95% CI 0.17, 0.39; OR = 0.31, 95% CI 0.16, 0.59, resp.). Additionally, females had lower odds of having injuries requiring medical care (OR = 0.73, 95% CI 0.66, 1.70). 

 Most groups had lower odds of having at least one chronic condition in comparison to NHW children with USB parents. In particular, FB children with both FB parents from “all other” ethnic groups were least likely to be in this category (OR = 0.10, 95% CI 0.05, 0.21). Moreover, Hispanic FB children with both FB parents and Hispanic and “all other” ethnic groups USB children with both FB parents followed with lower odds as well (OR = 0.33, 95% CI 0.22, 0.49; OR = 0.34, 95% CI 0.22, 0.49; and OR = 0.30, 95% CI 0.13, 0.65, resp.). As the age of children increased, the odds of having at least one chronic condition increased. Specifically, in the age 12–14 and 15–17 category, children were more than five times as likely to have at least one chronic condition (OR = 5.19, 95% CI 4.24, 3.36; OR = 5.42, 95% CI 4.42, 6.65, resp.). Females were less likely to have at least one chronic condition (OR = 0.61, 95% CI 0.55, 0.68). Children from poor families were almost three times as likely to have at least one chronic condition (OR = 2.84, 95% CI 2.41, 3.35) compared to those from families earning >400% FPL. 

## 4. Discussions

Our study is the first to characterize the significant and large differentials in health status characteristics among children from immigrant families. We have found that at the national level, wide disparities exist among the health measures we examined across racial/ethnic groups of similar immigrant family types. The adverse health profiles of Hispanic children of immigrant parents along with the large effect sizes are alarming, in areas of parent-reported physical and dental health, obesity/overweight, and injuries. 

Children of immigrant parents had few missed school days compared to native children, regardless of ethnicity. This is consistent with previous studies suggesting that this can be explained by the healthy immigrant effect, strong cultural values on education, or immigrant parents lacking resources to stay home with their children. The fact that FB children with both immigrant parents seemed to have a higher rate of injury that required medical attention compared with USB children may be an indication of less awareness of safety precautions.

Consistent with previous studies, children of immigrant parents have a higher rate of ever breastfeeding [17]. USB children of USB parents have a higher rate of having at least one chronic condition. However, since these chronic conditions had to be identified by a health care provider, this finding may be confounded by the differential access to health care by immigrant and nonimmigrant households. 

Child age, household income, and occasionally gender were also independent risk factors for most of the outcomes. Our data clearly illustrates that minority and immigrant status of parents conferred mixed effects on the health measures considered.

 In order to put our findings into perspective, it is important to consider the changing demographics of the US immigrant population. Close to one-quarter of US children have at least one FB parent. Immigrant family type is a complex variable, and the different combinations have been shown to confer differential risks on children's health care access and utilization outcomes [4, 9]. In addition to the differential eligibility for resources, immigrant status can also be a proxy for the length of residence in USA, English proficiency, as well as for the degree of acculturation, all factors that impact on children's health.

Some limitations of this analysis should be noted. The 2007 NSCH is conducted in English, Spanish, and four Asian languages, with the screener being in English or Spanish. This may bias the non-English respondents to be more educated and fluent in English, resulting in a likely underestimate of risk for the actual immigrant populations in USA. Undocumented immigrants who may be at the highest risk of adverse health are likely not to participate in the survey due to fear of exposing their illegal status, even though the survey contained no information on citizenship status. This selection bias likely excluded the most underserved populations. The increased use of cell phones may be yet introducing an additional source of bias for landline-only surveys [33, 34]. In addition, Asians who comprised four percent of the US population were collapsed into the “other” category in the public use data files. The “other” group with FB parents was likely Asians, while the ones with USB parents may include Native Americans, Hawaiians, and Pacific Islanders, leading to a lack of specificity when considering this major immigrant group. 

Our study examined multiple indicators of child health in a large, recent, nationally representative survey and clearly demonstrates the heterogeneous health status of children from immigrant households depending on the family's racial/ethnic background. Our findings should help improve provider awareness on specific issues and needs of these subpopulations. Outreach efforts by clinicians, public health professionals, and school systems are particularly needed to focus on improving physical and dental health, injury, and obesity prevention. Linguistic and culturally appropriate interventions should be targeted for specific immigrant families groups to mitigate such disparities.

## Figures and Tables

**Table 1:** US children's (0–17 yrs.) demographic and socioeconomic characteristics by immigrant family type. 2007 NSCH.

	Foreign-born child		US-born child/both immigrant parents		US-born child/one immigrant parent		US-born child/US-born parents	
	Weighted %	SE	Weighted %	SE	Weighted %	SE	Weighted %	SE
Total (unweighted)	2600		4622		2543		81767	
Child race/ethnicity								
Hispanic	56.5	2.3	64.7	1.8	36.7	2.8	12.6	0.4
Non-Hispanic white	15.1	1.4	5.7	0.5	39.6	2.6	63.5	0.4
Non-Hispanic black	11.2	1.7	6.8	0.7	6.7	1.1	15.3	0.3
Multirace	2.5	0.6	0.7	0.2	9.4	2.0	4.5	0.2
Other	14.7	1.5	22.2	1.6	4.5	1.6	4.15	0.2
Child age								
0 to 3	4.6	0.7	30.2	1.8	29.1	2.6	21.6	0.4
4 to 7	16.2	1.7	26.2	1.7	22.1	2.2	21.8	0.4
8 to 11	26.0	2.1	20.5	1.5	21.8	2.4	21.7	0.4
12 to 14	22.5	2.0	14.5	1.4	14.5	2.0	17.6	0.3
15 to 17	30.7	2.2	8.6	1.0	12.5	1.8	17.4	0.3
Child gender								
Male	50.0	2.4	51.6	1.9	53.6	2.7	51.0	0.4
Female	50.0	2.4	48.4	1.9	46.4	2.7	49.0	0.4
Family poverty level								
<100% FPL	39.4	2.3	35.5	1.9	11.6	2.2	15.9	0.3
100–199% FPL	23.7	2.1	26.4	1.7	19.0	2.4	20.3	0.4
200–399% FPL	20.6	1.9	19.7	1.6	30.7	2.4	32.9	0.4
≥400% FPL	16.3	1.5	18.4	1.2	38.7	2.6	30.8	0.4
Parent education								
Less than high school	31.7	2.4	36.9	1.9	10.0	2.4	7.6	0.2
High school	18.7	1.6	24.8	1.8	19.8	2.4	23.7	0.4
Greater than high school	44.4	2.3	37.6	1.8	70.0	2.9	60.5	0.4
Not stated	5.3	0.8	0.7	0.2	0.2	0.1	8.2	0.3
Primary language at home								
English	36.0	2.2	27.7	1.6	89.4	1.6	95.7	0.2
Other	64.0	2.2	72.3	1.6	10.6	1.6	4.3	0.2

All percentages are weighted.

All chi-square statistics for testing the association between immigrant status and each of the demographic characteristics were statistically significant at *P* < 0.0001, with the exception of gender.

**Table 2:** Selected health measures of children by race/ethnicity and immigrant family type. 2007 NSCH.

	Fair/poor health		Fair/poor dental health		Obese/overweight		Never breast-fed		Missed 11+ school days		Injury		At least one chronic condition	
	%	SE	%	SE	%	SE	%	SE	%	SE	%	SE	%	SE
*Hispanic *														
F-born child, both immigrant parents	17.2	3.3	37.2	3.4	36.0	7.5	21.8	7.5	3.6	1.3	12.4	5.5	7.5	1.4
US-born child, both immigrant parents	7.0	1.1	22.7	2.2	47.4	1.5	8.2	1.5	2.5	1.0	3.4	0.7	5.1	1.0
US-born child, one immigrant parent	5.1	2.5	16.1	5.7	41.9	5.0	17.3	5.0	4.8	3.0	12.2	4.2	8.8	2.9
US-born child, US-born parents	6.9	1.0	14.1	1.3	39.3	2.3	24.1	2.3	8.8	1.2	9.6	1.6	12.5	1.2
*Non-Hispanic White *														
One or both immigrant parents	0.8	0.3	3.9	0.8	27.9	4.5	10.1	1.6	4.2	1.0	11.2	2.9	7.3	1.1
US-born child, US-born parents	1.7	0.1	4.3	0.2	27.8	0.6	24.0	0.8	6.3	0.3	12.2	0.6	10.5	0.3
*Non-Hispanic Black *														
One or both immigrant parents	2.5	1.6	7.2	2.3	37.8	5.7	8.9	2.3	2.8	2.1	8.1	2.8	9.7	2.7
US-born child, US-born parents	4.6	0.5	9.5	0.7	41.4	1.5	48.2	2.0	4.5	0.5	8.4	1.5	14.6	0.8
*All other ethnic groups *														
F-born child, both immigrant parents	4.9	2.5	11.2	3.5	14.6	3.0	22.5	12.6	2.7	1.2	11.0	6.0	1.6	0.6
US-born child, both immigrant parents	3.0	1.7	7.8	2.6	18.8	5.2	11.7	3.2	0.3	0.1	4.2	1.3	3.0	1.1
US-born child, one immigrant parent	0.2	0.1	4.8	3.6	23.7	11.4	24.3	11.8	2.3	1.1	7.5	5.2	8.0	3.6
US-born child, US-born parents	3.1	0.4	7.4	0.7	35.4	2.1	25.4	25.6	6.6	0.9	12.6	1.7	12.2	0.9

**Table 3:** Adjusted odds of selected health measures among immigrant and US-born children aged 0–17 years.

	Fair/poor health			Fair/poor dental health			Obese/overweight 10–17 yrs			Never breast-fed 0–5 yrs			Miss school 11+ days 6–17 yrs			Injury requiring medical care 0–5 yrs			At least 1 chronic condition		
	OR	95% CI		OR	95% CI		OR	95% CI		OR	95% CI		OR	95% CI		OR	95% CI		OR	95% CI	
*Hispanic *																					
F-born child, both immigrant parents	6.88	4.17	11.37	10.10	7.22	14.12	1.14	0.73	1.79	0.49	0.21	1.15	0.36	0.17	0.78	1.03	0.38	2.80	0.33	0.22	0.49
US-born child, both immigrant parents	3.44	2.30	5.16	5.64	4.12	7.70	1.89	1.22	2.93	0.17	0.11	0.25	0.28	0.13	0.61	0.25	0.17	0.39	0.34	0.22	0.51
US-born child, one immigrant parent	2.86	1.01	8.16	3.87	1.58	9.48	1.81	0.86	3.84	0.58	0.30	1.11	0.64	0.17	2.37	0.99	0.45	2.18	0.78	0.38	1.58
US-born child, US-born parents	3.49	2.42	5.02	3.25	2.54	4.17	1.58	1.25	2.01	0.79	0.60	1.03	1.22	0.88	1.70	0.77	0.52	1.14	1.04	0.82	1.30
*Non-Hispanic White *																					
One or both immigrant parents	0.52	0.27	1.00	1.00	0.61	1.64	1.12	0.76	1.65	0.39	0.27	0.56	0.71	0.45	1.14	0.86	0.49	1.53	0.72	0.51	1.03
US-born child, US-born parents	1.00		Reference	1.00		Reference	1.00		Reference	1.00		Reference	1.00		Reference	1.00		Reference	1.00		Reference
*Non-Hispanic Black *																					
One or both immigrant parents	1.24	0.33	4.57	1.66	0.76	3.61	1.53	0.93	2.52	0.25	0.14	0.46	0.38	0.08	1.74	0.65	0.31	1.40	0.79	0.42	1.47
US-born child, US-born parents	1.75	1.32	2.34	1.67	1.37	2.03	1.59	1.37	1.83	2.19	1.82	2.63	0.53	0.42	0.68	0.67	0.45	1.00	1.05	0.91	1.20
*All other ethnic groups *																					
F-born child, both immigrant parents	2.53	0.79	8.08	2.93	1.26	6.82	0.44	0.28	0.71	0.93	0.22	3.96	0.37	0.15	0.90	0.95	0.30	3.03	0.10	0.05	0.21
US-born child, both immigrant parents	2.07	0.63	6.82	1.92	0.92	4.02	0.57	0.29	1.09	0.42	0.22	0.80	0.04	0.01	0.12	0.31	0.16	0.59	0.30	0.13	0.65
US-born child, one immigrant parent	0.11	0.02	0.50	1.25	0.30	5.21	0.76	0.25	2.29	0.96	0.32	2.82	0.38	0.14	1.01	0.59	0.14	2.55	0.94	0.39	2.26
US-born child, US-born parents	1.57	1.15	2.15	1.61	1.27	2.04	1.38	1.14	1.66	0.93	0.76	1.15	0.97	0.71	1.33	1.05	0.76	1.45	1.10	0.93	1.31
*Age, y *																					
0 to 3	1.00		Reference	1.00		Reference													1.00		Reference
4 to 7	2.36	1.69	3.30	3.84	2.83	5.22							1.00		Reference				3.13	2.55	3.84
8 to 11	2.71	1.99	3.67	5.37	3.95	7.3							1.09	0.84	1.41				4.97	4.08	6.06
12 to 14^∧^	3.57	2.57	4.96	3.73	2.71	5.14	1.00		Reference				1.43	1.11	1.85				5.19	4.24	6.36
15 to 17^∧∧^	3.21	2.36	4.36	2.85	2.03	4.02	0.60	0.54	0.67				1.55	1.23	1.97				5.42	4.42	6.65
*Gender *																					
Male	1.00		Reference	1.00		Reference	1.00		Reference	1.00		Reference	1.00		Reference	1.00		Reference	1.00		Reference
Female	0.83	0.66	1.04	0.83	0.72	0.97	0.74	0.66	0.82	0.96	0.84	1.09	1.03	0.86	1.23	0.73	0.66	0.88	0.61	0.55	0.68
*Household poverty status (ratio of family income to poverty threshold) *																					
Below 100%	5.09	3.50	7.40	7.53	5.87	9.66	2.38	1.99	2.85	3.03	2.47	3.72	2.62	2.00	3.43	0.90	0.67	1.21	2.84	2.41	3.35
100–200%	2.77	1.87	4.10	4.86	3.75	6.31	1.93	1.65	2.26	1.95	1.61	2.37	1.92	1.49	2.48	0.86	0.64	1.16	2.07	1.77	2.44
200–400%	1.09	0.72	1.64	2.11	1.64	2.71	1.47	1.29	1.68	1.41	1.19	1.68	1.34	1.03	1.73	0.82	0.65	1.04	1.31	1.13	1.52
At or above 400%	1.00		Reference	1.00		Reference	1.00		Reference	1.00		Reference	1.00		Reference	1.00		Reference	1.00		Reference

OR: odd ratio; SE: standard error; CI: confidence interval. F-born: foreign-born.

^∧^The reference group for obesity/overweight aged 10–13.

^∧∧^Age group 14–17 for obesity/overweight measure.

## Abbreviations

OR: Odds ratio
CI: Confidence interval.
